# Multistage investigation of predictive factors for tracheostomy in brain injury: a bibliometric, descriptive, and retrospective analysis

**DOI:** 10.3389/fneur.2025.1648046

**Published:** 2025-09-24

**Authors:** Haotian Wu, Hongyue Wang, Xue Yang, Lihua Jin, Qian Liu, Yao Zhou, Zihan Chen, Liqing Yao

**Affiliations:** ^1^Department of Rehabilitation Medicine, The Second Affiliated Hospital of Kunming Medical University, Kunming, China; ^2^Department of Radiology, The Second Affiliated Hospital of Kunming Medical University, Kunming, China

**Keywords:** brain injury, traumatic brain injury, tracheostomy, critical care, neurocritical care

## Abstract

**Background:**

Brain injury, particularly traumatic brain injury (TBI), stands as a prominent global cause of mortality and disability. Tracheostomy in TBI patients may lead to added complications. However, the current literature lacks consistency regarding predictive factors for tracheostomy in this patient population. This study seeks to investigate and validate specific predictive factors associated with the need for tracheostomy in TBI patients through a multi-faceted approach involving bibliometric analysis, descriptive examination, and retrospective research.

**Methods:**

This study employs a multi-stage design: a bibliometric analysis of recent literature on tracheostomy predictors in brain injury patients, followed by a descriptive analysis using PRISMA 2020 guidelines. Clinical data from TBI patients are collected, with univariate and Spearman correlation analyses identifying independent predictive factors.

**Result:**

The bibliometric analysis reveals growing research on tracheostomy prediction in brain injury patients, with key themes including “mortality,” “management,” and “outcomes.” Descriptive analysis of five studies identified common predictors such as low Glasgow Coma Scale (GCS) score, advanced age, multiple injuries, pulmonary complications, and brain imaging features. Retrospective clinical data showed a significant association between diffuse axonal injury (DAI) and tracheostomy need, particularly with injury causes and decompressive craniectomy. Spearman correlations highlighted significant relationships with GCS, illness duration, age, pupil response, Marshall score, and brainstem injury, as well as weak correlations with DAI and injury causes.

**Conclusion:**

This study identified predictive factors for tracheostomy in brain-injured patients, focusing on TBI. Key factors include GCS score, DAI presence, age, decompressive craniectomy, and injury severity.

## 1 Introduction

Brain injury significantly impacts patients’ physical and mental health, placing a substantial burden on both their families and society (1, 2). Its treatment has always been a focus of attention. Brain injury covers a variety of causes, including traumatic brain injury (TBI), stroke, and subarachnoid hemorrhage (SAH). Among these, TBI stands out as a primary contributor to global mortality and disability (3, 4).

Patients with brain injuries often require many treatments such as decompressive craniectomy and tracheotomy due to medical conditions. Tracheotomy is a critical intervention to establish a reliable artificial airway, particularly for patients needing prolonged mechanical ventilation or facing challenges with extubation, as well as in cases of upper airway obstruction (5). Despite being an advanced treatment option, tracheotomy is associated with significant complications ranging from mild issues like bleeding and subcutaneous emphysema to severe conditions such as pneumothorax, wound infection, tracheoesophageal fistula, tracheal stenosis, and granulation tissue formation. These complications can hinder patient recovery, leading to extubation difficulties, recurrent infections, or life-threatening situations (6–8).

Patients with brain injuries requiring tracheotomy typically have critical conditions, prolonged mechanical ventilation, or severe underlying diseases (9, 10). These individuals are already on the verge of respiratory function decompensation, facing a higher risk of respiratory failure and increased fragility in their overall physiological reserve. Early monitoring of patients likely to need tracheotomy can help identify those at increased risk of respiratory failure, allowing for timely interventions and preventive measures to reduce postoperative complications and improve prognosis.

Despite the numerous studies investigating the predictive factors for tracheostomy in brain injury patients, a unified, accurate, and universally applicable standard has not been established. This study aims to systematically assess current research, identify trends and future directions, and explore predictive factors for tracheostomy in specific brain injury types, such as TBI.

Initially, a bibliometric analysis was carried out on recent literature to pinpoint crucial research trends and gaps in the current understanding of brain injury and tracheostomy needs. Secondly, we aimed to quantitatively integrate the predictive factors reported across existing studies through a meta-analysis to obtain more reliable effect estimates. However, due to substantial heterogeneity in study designs, variations in patient characteristics, and diverse outcome measures used, we refined our approach by selecting and systematically reviewing the literature following the PRISMA 2020 guidelines to ensure methodological transparency and rigor throughout the research process. Lastly, to explore in greater detail the relationship between disease severity in brain injury patients, particularly those with TBI, and the need for tracheostomy, we retrospectively gathered clinical data from TBI patients for analysis. We hypothesize that the severity of brain injury is the primary factor determining the necessity for tracheostomy, regardless of whether they have pneumonia, chest injuries, or other factors.

## 2 Methods

**Figure 1:**
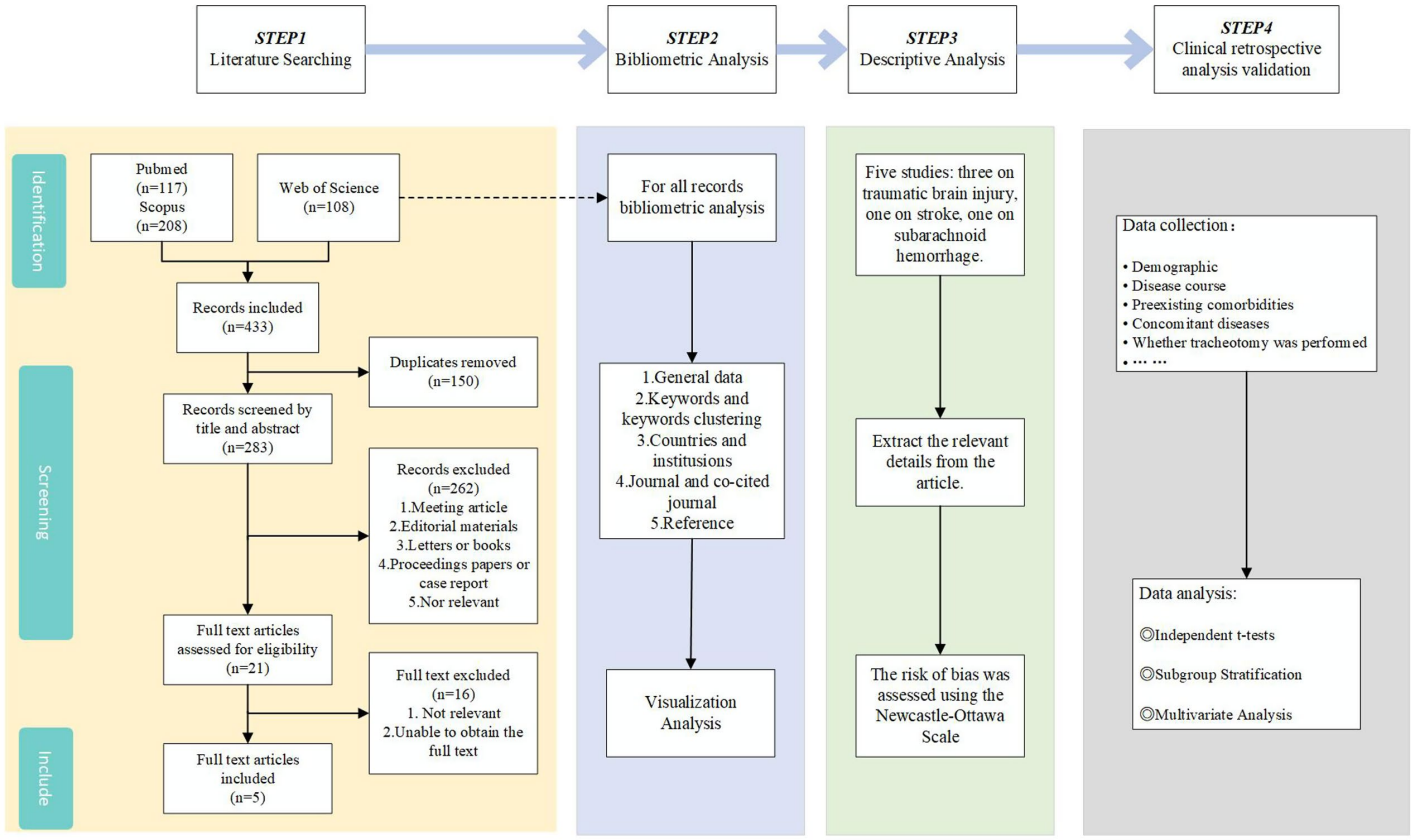
Study selection flowchart and methodology summary.

To validate our hypothesis, we conducted a three-step study (see Figure 1). Firstly, we conducted a bibliometric analysis of recent literature to comprehend research trends and key issues in the field of tracheostomy for brain injury patients. Secondly, we carried out a systematic review of existing literature following the PRISMA 2020 guidelines and conducted a descriptive analysis to summarize the main findings of each study, identifying specific factors. Lastly, we performed a retrospective analysis of clinical data from TBI patients to deeply investigate the relationship between disease specificity in brain injury patients and the necessity for tracheostomy, aiming to confirm the alignment of our hypothesis with the results of the data analysis.

### 2.1 Retrieval strategy

A literature search was performed in the Web of Science, PubMed, and Scopus databases utilizing the following search terms: “(tracheotomy OR tracheostomy OR percutaneous tracheotomy OR emergency tracheotomy OR surgical tracheotomy) AND (Predictive Factors OR predictors OR prediction OR Predictive OR Indications) AND (Brain injury OR Acute Brain Injury OR Stroke OR Traumatic brain injury OR Cerebral injury OR Head Injury).” Two authors independently assessed the search results according to predetermined inclusion criteria. In instances of disagreement, a third author was involved to achieve consensus. A detailed account of the screening process is delineated in Figure 1.

### 2.2 Inclusion and exclusion criteria

For increased precision, we established the following inclusion criteria: studies had to involve patients with brain injury and focus on predictive factors of tracheostomy. In contrast, irrelevant studies, experimental research, non-English articles, and studies with inaccessible data were excluded.

### 2.3 Bibliometric analysis

Bibliometric analysis in this study is limited to data integration solely from Web of Science due to current constraints. The selected articles were imported into CiteSpace (version 6.2.R7) and VOSviewer (version 1.6.20) software for organization and analysis. Basic information, including authors, institutions, keywords, annual publication volume, countries, and regions of publication, was extracted. Keyword co-occurrence and clustering analysis, along with article co-citation analysis, were performed. Visualization of the data was carried out using CiteSpace, R software, VOSviewer, and Origin software.

### 2.4 Descriptive analysis

Due to the limited number of studies (*n* = 5) and high heterogeneity (involving TBI, SAH, and stroke), a meta-analysis was not feasible. Instead, a thematic descriptive synthesis was conducted, extracting data on Author, Country and Institution, Year, Type of Brain Injury, Strong Predictors, Age, Total Number of Patients, Inclusion Criteria, Exclusion Criteria, Type of Study, Assessments, Interventions, Primary Outcomes, and Secondary Outcomes from each of the five articles. The risk bias assessment utilized the Newcastle-Ottawa Scale.

### 2.5 Clinical retrospective analysis validation

To further validate our study, 80 patients with traumatic brain injury, aged 18 to 70 and with a disease duration of up to 30 days, were retrospectively analyzed at a university-affiliated hospital. The data included demographics, medical history, clinical scores (Marshall score, AIS head score), and treatment details such as tracheotomy. Statistical analysis was conducted using SPSS 30.0, employing Mann–Whitney and Wilcoxon tests for intergroup and intragroup comparisons, respectively. Spearman correlation analysis was used to assess relationships, with statistical significance set at *p* < 0.05.

## 3 Result

### 3.1 Bibliometric analysis

#### 3.1.1 Bibliometric analysis of keywords AND keywords clustering

**Figure 2:**
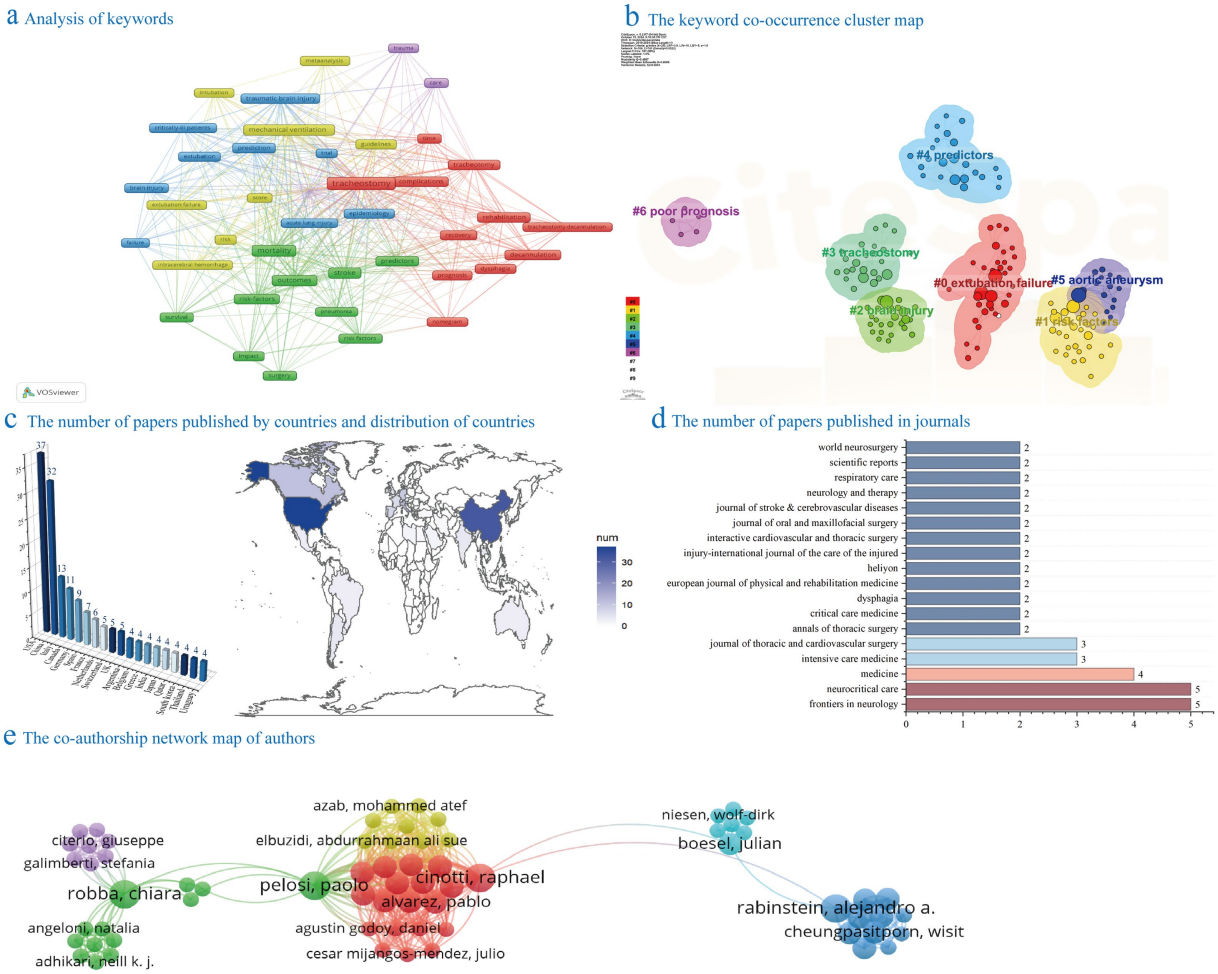
Bibliometric analysis. Panel **(a)** displays the frequency chart of keywords in the field of predicting the necessity for tracheostomy in patients with brain injuries from September 2, 2019, to September 2, 2024. Panel **(b)** illustrates the keyword clustering chart in the same field during the same period. Panel **(c)** shows the bar chart and topographic map of the number of papers published by countries in the field of predicting the need for tracheostomy in patients with brain injury from September 2, 2019, to September 2, 2024. Panel **(d)** exhibits the bar chart displaying the number of papers published in journals within the same field and time frame. Panel **(e)** visualizes the collaborative relationships among authors who have published articles concerning the prediction of tracheostomy necessity in patients with brain injuries between September 2, 2019, and September 2, 2024.

Through statistical analysis of keywords, we examined the topic distribution and research trends in predicting tracheostomy. A total of 591 keywords were identified, with “Tracheostomy,” “mortality,” “management,” “outcomes,” “mechanical ventilation,” “stroke,” “complications,” “traumatic brain injury,” “predictors,” and “prediction” being the top 10 most frequently used keywords (see Figure 2a). Figure 2b illustrates the keyword clustering analysis, categorizing keywords into seven clusters: “extubation failure, risk factors, brain injury, tracheostomy, predictors, aortic aneurysm, and poor prognosis.” While exploring the occurrence of keywords, we found that the intensities were all relatively small and did not warrant further discussion, so we ignored them.

#### 3.1.2 Bibliometric analysis of authors

Analysis of author-related data and author collaboration graphs provide insights into research distribution and collaboration patterns in tracheostomy prediction. Supplementary Table 2 indicates that several authors have published 3–4 articles in this field, with GIRARDI LN, GAMBARDELLA, and LAU having the most publications at 4 each. ASEHNOUNE K has the highest number of citations with 113. The author collaboration network analysis in Figure 2e identifies main research groups like PELOSI et al., CINOTTI et al., and RABINSTEIN et al. It is important to note that software-based bibliometrics calculate individual authors separately, potentially introducing bias due to multiple authors contributing to a single article. It should be noted that since one article may be completed by multiple authors, individual authors are calculated separately in software-based bibliometrics, which may lead to potential bias.

#### 3.1.3 Bibliometric analysis of countries and institutions

The analysis of publication numbers from various institutions and countries identified the geographic distribution and research concentration in the field. Figure 2c depicts countries with more than four publications, showing the United States, China, and Italy as the top contributors with 37, 32, and 13 publications, respectively. Supplementary Table 3 highlights key institutions, with the University of Toronto leading in publications (7) and averaging 8.1 citations per article. Notably, the University of Genoa stood out with an average citation count of 21.6 per publication, indicating high research quality and impact. Other active contributors include Capital Medical University, University Health Network, Huazhong University of Science and Technology, and Weill Cornell Medical College, each providing valuable research outcomes in the field.

#### 3.1.4 Bibliometric analysis of journals

In the realm of tracheostomy prediction, a thorough examination of journal-specific data can unveil the trends in dissemination and the landscape of research impact. Figure 2d displays journals that have published more than 2 papers, highlighting “Frontiers in Neurology” and “Neurocritical Care” as the most prolific with 5 related publications each. Supplementary Table 4 presents the most pertinent and highly cited journals, with impact factors ranging from 3.1 to 63.1 in 2023. The top three journals based on citation count are Stroke (130), Critical Care Medicine (127), and Neurocritical Care (97).

#### 3.1.5 Bibliometric analysis of references

The number of citations in tracheostomy research literature is a pivotal indicator of a study’s impact and significance. Supplementary Table 5 presents the top five most cited studies, with notable contributions from Bösel et al., Coplin et al., and Pelosi et al. (11–13), focusing on early tracheostomy in stroke patients, delayed extubation effects in brain-injured patients, and mechanical ventilation management in neurocritical care patients.

### 3.2 Descriptive analysis

The five studies analyzed three distinct disease populations as outlined in Supplementary Table 1. Specifically, three studies centered on traumatic brain injury, comprising two retrospective studies and one prospective study involving a total of 2,586 patients. Additionally, one study conducted a prospective investigation with 635 stroke patients, while another retrospective study examined 488 patients with subarachnoid hemorrhage.

#### 3.2.1 Predictive factors for tracheostomy in traumatic brain injury patients

The ISS score was assessed in all three studies, revealing significant inter-group differences. Chiara et al. reported mean ISS scores of 33.45 and 38.4 (*p* < 0.001) (14). In the study of Felix et al. (15), ISS scores were 26.2 ± 12.0 and 36.0 ± 12.0 (*p* < 0.001). Ryne et al. (16) documented ISS standard deviations of 27 and 33 (*p* < 0.001). Nonetheless, logistic regression analyses across these studies did not reveal significant correlations.

Two studies demonstrated a significant correlation between a lower Glasgow Coma Scale score (GCS ≤ 8) and the necessity for tracheostomy. Chiara et al. (16) reported a hazard ratio (HR) of 1.51–1.98 (*p* < 0.001). In a separate study, Ryne et al. identified an odds ratio (OR) of 0.52 (95% CI [0.4, 0.68], *p* < 0.001).

Furthermore, the severity of TBI and the presence of comorbidities significantly impact the necessity for tracheostomy. Specifically, chest trauma (HR = 1.24, 95% CI = 1.01–1.52, *p* = 0.020) and abnormal pupil response (lack of reaction in at least one pupil, HR = 1.63–1.96, *p* < 0.001) were identified as significant factors increasing the likelihood of tracheostomy requirement (14). The AISThorax score (*p* < 0.001) and AISHead ≥ 3 (OR = 4.149, 95% CI [2.967–5.803], *p* < 0.001) were also significantly linked to the need for tracheostomy (15). While the Marshall score yielded a *p*-value of 0.02 in intergroup comparisons, the odds ratio (OR) in logistic regression analysis stood at 1.30 (95% CI [0.97–1.74], *p* = 0.074), indicating a lack of strong correlation (16).

In terms of post-injury complications and treatment, pre-hospital intubation (AOR = 2.494, 95% CI [1.412–4.405], *p* < 0.001), pneumonia diagnosed during ICU stay (AOR = 4.374, 95% CI [2.503–7.642], *p* < 0.001), and mechanical ventilation duration (AOR = 1.008/h, 95% CI [1.006–1.009], *p* < 0.001) were significantly correlated with the necessity for tracheostomy (15, 16). The frequency of visits to the operating room (AOR = 1.75, 95% CI [1.04–2.97], *p* = 0.036), reintubation (AOR = 8.45, 95% CI [1.91–37.44], *p* = 0.005), and external ventricular drain (EVD) insertion (AOR = 3.48, 95% CI [1.27–9.58], *p* = 0.016) were also linked to the need for tracheostomy (16). Additionally, Chiara et al. (14) demonstrated a significant association between respiratory system complications, such as respiratory failure (47.8% vs. 24.2%, *p* < 0.001) and ventilator-associated pneumonia (35.5% vs. 14.0%, *p* < 0.001), and the requirement for tracheostomy.

Additionally, the patient’s general condition can influence the decision regarding tracheostomy. Research conducted by Chiara et al. demonstrated a significant association between advanced age and tracheostomy. Notably, with every 5-year increase in age, there was a 4% higher risk of tracheostomy (HR = 1.04, 95% CI [1.01–1.07], *p* = 0.003) (14).

#### 3.2.2 Predictive factors for tracheostomy in subarachnoid hemorrhage patients

In patients with subarachnoid hemorrhage who underwent tracheostomy, age ≥60 years (OR: 3.79, 95% CI [1.56–9.44], *p* = 0.004), hypertension (OR: 3.23, 95% CI [1.62–6.44], *p* = 0.001), high neutrophil-to-lymphocyte ratio (OR: 3.26, 95% CI [1.24–8.60], *p* = 0.017), high platelet-to-lymphocyte ratio (OR: 2.66, 95% CI [1.23–5.76], *p* = 0.013), low lymphocyte-monocyte ratio (OR: 6.34, 95% CI [3.18–12.66], *p* < 0.001), high systemic inflammatory response index (OR = 9.56, 95% CI [4.63–19.75], *p* < 0.001), high WFNS grade (OR = 7.91, 95% CI [2.62–23.84], *p* < 0.001), high mFisher grade (OR: 5.95, 95% CI [2.05–17.26], *p* < 0.001) and high BNI grade (OR: 11.91, 95% CI [4.17–33.97], *p* < 0.001), large aneurysm (OR: 2.42, 95% CI [1.08–5.41], *p* = 0.032), long operation time (OR: 3.01, 95% CI [1.54–5.88], *p* = 0.001) (17).

#### 3.2.3 Predictive factors for tracheostomy in stroke patients

The incidence of hospital-acquired pneumonia (HAP) was significantly higher (OR = 21.26, 95% CI = 2.76–163.56, *p* = 0.003). Additionally, the failure of extubation (OR = 8.41, *p* < 0.001), decompressive craniectomy (OR = 9.94, 95% CI = 3.92–25.21, *p* < 0.001), and sepsis (OR = 5.39, 95% CI = 1.71–16.91, *p* = 0.004) were noted (18).

### 3.3 Clinical retrospective analysis validation for patients with traumatic brain injury

This study involved 82 patients with brain injuries, aged between 18 and 70 years, with a mean age of 42.85 ± 14.23. The cohort comprised 60 males and 22 females. The illness duration varied from 0 to 30 days, with a mean of 16.45 ± 10.71 days. Of the patients, 58 had non-diffuse axonal injuries, and 24 had axonal injuries. Furthermore, 38 patients did not undergo tracheotomy, while 44 patients did.

#### 3.3.1 Analyze diffuse axonal injury as a factor

No statistically significant differences in baseline characteristics, such as age and sex, were observed between patients with and without DAI. However, significant disparities were found in tracheostomy (*p* = 0.013) and injury etiology (*p* = 0.007) when comparing the groups based on the presence of DAI, while other factors (e.g., spinal cord injury, thoracic injury, pneumonia) demonstrated no statistical significance (Table 1).

**Table 1:** Comparative analysis of groups with and without diffuse axonal injury.

Item		Diffuse axonal injury		*p* value
		No	Yes	
Age		46 (18,70)	49 (18,62)	0.779
Gender	Female	45	15	0.163
	Male	13	9	
Duration of injury		20 (0,30)	20 (0,30)	0.174
Cause of injury	Injured by falling	1	1	**0.007** ^*^
	Fall injury	34	5	
	Car accidents	23	18	
GCS gread	<8	21	10	0.645
	>8	37	14	
Abnormal pupil response	No	51	18	0.147
	Yes	7	6	
Marshall gread	1	3	2	0.918
	2	27	11	
	3	11	4	
	4	12	2	
	5	3	0	
	6	2	5	
AISHead gread	<3	4	1	0.640
	≥3	54	23	
AISThora gread	<3	48	17	0.228
	≥3	10	7	
Tracheostomy	No	32	6	**0.013** ^*^
	Yes	26	18	
Tracheostomy after injury		7 (0,17)	5 (0,30)	0.747
Duration of tracheostomy		35 (24,90)	47.5 (15,249)	0.452
Hypertension	No	54	24	0.190
	Yes	4	0	
Diabetes	No	52	21	0.778
	Yes	6	3	
Copd	No	58	24	1.000
	Yes	0	0	
Epilepsy	No	55	23	0.848
	Yes	3	1	
Coronary heart disease	No	57	24	0.520
	Yes	1	0	
Pneumonia	No	24	8	0.499
	Yes	34	16	
Decompressive Craniectomy	No	33	16	0.415
	Yes	25	8	
Spinal cord injury	No	56	24	0.360
	Yes	2	0	
Rib fractures	No	43	15	0.295
	Yes	15	9	
Brain stem injury	No	55	21	0.249
	Yes	3	3	
Skull fracture	No	26	13	0.444
	Yes	32	11	

*indicates statistical significance.

#### 3.3.2 Sub-group analysis

In tracheostomized patients with traumatic brain injury, when comparing groups based on the presence of diffuse axonal injury, significant differences were found in the cause of injury (*p* = 0.019) and whether decompressive craniectomy was performed (*p* = 0.025). In non-tracheostomized TBI patients, no statistically significant differences were observed when comparing groups based on diffuse axonal injury (see Table 2).

**Table 2:** Comparative analysis of patients with and without tracheostomy.

Item		Non-tracheostomy		*p* value	Tracheostomy		*p* value
		Diffuse axonal injury			Diffuse axonal injury		
		No	Yes		No	Yes	
Age (years)		41 (18,68)	29 (0,21)	0.052	52 (23,70)	53 (18,62)	0.971
Gender	Female	26	4	0.428	19	11	0.408
	Male	6	2		7	7	
Duration of injury (days)		12 (0,30)	14 (0,21)	1.000	22 (0,29)	24 (0,30)	0.292
Cause of injury	Injured by falling	0	0	0.245	1	1	0.019^*^
	Fall injury	19	2		15	3	
	Car accidents	13	4		10	14	
GCS gread	<8	4	0	0.366	17	10	0.515
	>8	28	6		9	8	
Abnormal pupil response	No	30	6	0.535	21	12	0.294
	Yes	2	0		5	6	
Marshall gread	1	3	0	0.691	0	2	0.519
	2	17	4		10	7	
	3	7	0		4	4	
	4	5	2		7	0	
	5	0	0		3	0	
	6	0	0		2	5	
AISHead gread	<3	4	0	0.366	0	1	0.229
	≥3	28	6		26	17	
AISThora gread	<3	28	5	0.785	20	12	0.458
	≥3	4	1		6	6	
Decompressive craniectomy	No	26	5	0.905	7	11	0.025^*^
	Yes	6	1		19	7	
Brain stem injury	No	32	6	1.000	23	15	0.630
	Yes	0	0		3	3	

*indicates statistical significance.

#### 3.3.3 Correlation analysis

**Figure 3:**
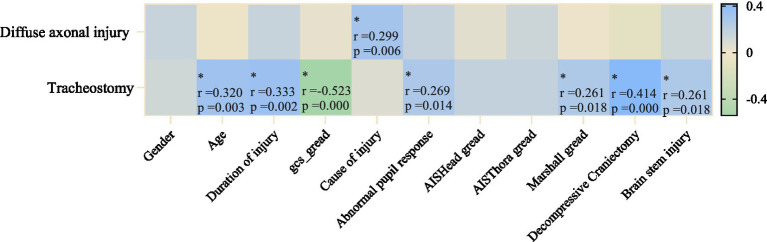
Correlation analysis diagram. *indicates statistical significance.

To further clarify the related factors of tracheostomy in TBI patients, we performed Spearman correlation analysis between diffuse axonal injury and tracheostomy with factors such as injury severity, time from onset to admission, and injury cause (see Figure 3). We found that GCS score (r = −0.523, *p* < 0.001) was negatively moderately correlated, while whether decompressive craniectomy was performed (r = 0.414, *p* < 0.01), course of disease (r = 0.333, *p* = 0.02), and age (r = 0.320, *p* = 0.003) were positively moderately correlated with tracheostomy. Pupillary abnormal reaction (r = 0.269, *p* = 0.014), Marshall score (r = 0.261, *p* = 0.018), and Brain stem injury (r = 0.261, *p* = 0.018) showed positive weak correlation with tracheostomy requirement. The cause of injury was positively weakly correlated with diffuse axonal injury (r = 0.299, *p* = 0.006).

## 4 Discussion

A bibliometric analysis was initially conducted to review recent research on predictive factors for tracheostomy in brain-injured patients. Subsequently, a meta-analysis was performed to identify key predictive factors. Due to study design heterogeneity and patient population variations, a quantitative meta-analysis was not feasible. Instead, a descriptive analysis was undertaken to summarize the main findings and highlight factors influencing tracheostomy decisions. To delve deeper into the relationship between disease specificity and tracheostomy requirements in brain-injured patients, a retrospective analysis of clinical data from TBI patients was conducted. The analysis revealed associations between tracheostomy requirements and factors such as diffuse axonal injury, GCS score, performance of decompressive craniectomy, disease course, age, pupillary response abnormalities, Marshall score, and brain stem injury. Moreover, age and TBI severity were found to be correlated with tracheostomy, particularly injury severity.

### 4.1 Current status of research

This study utilizes bibliometric methods to examine research trends and key issues in the field of tracheostomy for brain-injured patients. The analysis indicates a consistent growth in research over the past 5 years, with an annual publication volume exceeding 20 papers, reflecting sustained interest in predictive factors related to tracheostomy. Despite data gaps in 2019 and 2024 potentially impacting trend analysis, an overall upward trajectory is observed. The study identifies prominent authors such as GIRARDI LN, GAMBARDELLA, and LAU as leading figures in this field, with ASEHNOUNE K emerging as a highly influential author, with 113 citations. Geographically, the United States, China, and Italy lead in research output, with a concentration of studies in these regions. The University of Toronto stands out among institutions, leading in both publication volume and citation counts, highlighting its research expertise in tracheostomy studies. Frontiers in Neurology and Neurocritical Care are identified as primary journals for publishing tracheostomy-related literature. Keyword analysis indicates that “Tracheostomy” is the predominant theme, frequently associated with terms like “mortality,” “management,” and “outcomes,” underscoring the emphasis on patient survival, treatment management, and prognosis. Cluster analysis reveals seven key themes, particularly focusing on “extubation failure” and “brain injury,” shedding light on the correlation between extubation failure and the decision-making process regarding tracheostomy, as well as the distinct challenges in tracheostomy decision-making for patients with brain injuries. Overall, the research primarily centers on the implementation of tracheostomy, its application in various clinical scenarios, and the evaluation of patient prognosis, providing valuable insights for more accurate decision-making and enhanced patient outcomes.

### 4.2 Descriptive analysis

#### 4.2.1 Predictive factors for tracheostomy in traumatic brain injury patients

The severity of head injury and its scoring play a crucial role in predicting the need for endotracheal intubation in patients with TBI. Specifically, the lack of pupil response upon hospital admission is positively correlated with the requirement for endotracheal intubation. Pupil response, a straightforward and intuitive indicator of the nervous system, often indicates the presence of severe head injury (19, 20).

Furthermore, we found that, besides the GCS score, various injury severity scores correlated with the necessity for tracheostomy. Specifically, the Injury Severity Score (ISS) exhibited a strong association with tracheostomy requirement in TBI cases, displaying significant intergroup variations. The ISS was initially introduced by Baker et al. in the 1970s as a tool for trauma severity assessment (21), subsequently becoming a widely utilized standard among trauma specialists (22). Research by Foreman et al. (23) indicated that, compared to singular neurological assessments like the GCS, the ISS displayed heightened sensitivity in predicting post-traumatic outcomes and respiratory issues. In a study, the ISS was identified as a crucial factor in forecasting early tracheostomy needs (24). Nevertheless, our analysis revealed that the ISS did not independently exhibit statistically significant predictive capability in logistic regression analysis (14–16), potentially attributable to multicollinearity within the model, where the ISS score’s correlation with other variables (e.g., age and initial GCS score) weakened its autonomous predictive capacity. Notably, the ISS score has inherent limitations, primarily assessing injury severity in the three most affected body regions based on anatomical damage, thereby inadequately reflecting the patient’s physiological and neurological status. Therefore, several scoring systems, including the New Injury Severity Score (NISS), Trauma and Injury Severity Score (TRISS), and Revised Trauma Score (RTS), have been developed and shown to have superior predictive accuracy in specific contexts (25–27). Additionally, the AIS HEAD score, utilized for characterizing head injuries, correlates positively with an elevated risk of tracheostomy. AIS HEAD is a specialized scoring system for assessing the location and severity of brain injuries, enabling a more precise evaluation of the extent of injury and detection of brain stem involvement. A study by Jiang et al. involving 846 severe TBI patients clearly demonstrated a strong association between a high AIS Head score and unfavorable outcomes (28). Thus, a high AIS Head score may serve as an indicator of central respiratory suppression.

The need for tracheostomy in TBI patients is closely associated with specific comorbidity, complications, and treatment. TBI patients commonly present with thoracic trauma, which can result in airway obstruction, lung function impairment, or precipitate acute respiratory distress syndrome (ARDS). ARDS is linked to high mortality rates and is a crucial factor to consider when deciding on tracheostomy placement (29, 30). This aligns with previous research emphasizing the comprehensive management of polytrauma patients (31, 32). Ostermann et al. (33), in their study on elderly TBI patients, identified severe thoracic trauma as a significant independent predictor of poor prognosis and advocated for active respiratory support in such cases. Pneumonia, a common complication, impacts the tracheostomy needs of TBI patients. Factors such as impaired consciousness, diminished cough reflex, and swallowing difficulties render TBI patients highly susceptible to aspiration and respiratory infections, elevating the likelihood of requiring a tracheostomy (34). Tracheostomy necessity is associated with pre-hospital intubation, prolonged mechanical ventilation, and surgical factors (e.g., increased operating room transfers, reintubation, and external ventricular drain placement). Prolonged mechanical ventilation can lead to complications like ventilator-associated pneumonia, prompting clinicians to opt for tracheostomy to enhance airway management and facilitate ventilator weaning (35, 36). The occurrence of multiple operating room transfers, reintubation, and external ventricular drain insertion during surgery is closely correlated with heightened tracheostomy needs, likely due to elevated anesthesia and surgical risks, as well as the potential for infections and other respiratory complications (37). While pre-hospital intubation aids in maintaining respiratory function in critically ill patients, constraints in pre-hospital settings may result in airway injuries or infections, indirectly necessitating tracheostomy (38, 39). Therefore, comprehensive management of TBI patients should encompass measures to mitigate pre-hospital delays, conduct thorough assessments, enhance complication control during intensive care, and optimize surgical procedures to enhance patient outcomes.

#### 4.2.2 Predictive factors for tracheostomy in subarachnoid hemorrhage patients

Several biochemical and clinical indicators are closely associated with the requirement for tracheostomy in cases of subarachnoid hemorrhage. The findings of this investigation indicate that age ≥ 60 years, hypertension, specific hematological markers (elevated neutrophil-to-lymphocyte ratio, increased platelet-to-lymphocyte ratio, reduced lymphocyte-monocyte ratio, elevated systemic inflammatory response index), and various neurological grading systems (WFNS, mFisher, BNI grading) are all linked to a heightened risk. Moreover, an extended duration of operation and larger size of arterial aneurysm are also positively correlated with the necessity for tracheostomy (17).

#### 4.2.3 Predictive factors for tracheostomy in stroke patients

A systematic analysis in stroke patients revealed a significantly higher incidence of HAP among those who underwent tracheotomy. Failed extubation, debridement and decompressive craniectomy, and sepsis were all positively associated with tracheotomy, highlighting an elevated infection risk post-procedure (18). These findings emphasize the importance of rigorous infection monitoring and prevention strategies during treatment, underscoring the need to optimize clinical interventions to mitigate complications’ adverse impact on prognosis.

### 4.3 Clinical retrospective analysis validation for patients with traumatic brain injury

Patients necessitating airway maintenance or long-term mechanical ventilation often require tracheotomy, with common indications being severe pulmonary infections, multiple traumas, and conditions resulting in respiratory failure. Two primary types of tracheotomy procedures are currently employed: surgical tracheotomy and percutaneous dilatational tracheotomy, with the latter being the preferred technique due to its minimal invasiveness and operational convenience (40, 41).

In our analysis, we identified specific predictive factors for tracheostomy necessity in patients with different brain injuries. For TBI patients, the need for tracheostomy is associated with injury severity, trauma score, complications, and treatment course. Clinical validation, however, revealed no significant link between tracheostomy necessity and factors like AIS Throat, pneumonia, or mechanical ventilation duration. Conversely, indicators of brain injury severity such as diffuse axonal injury, GCS score, decompressive craniectomy, disease duration, age, abnormal pupil response, Marshall score, and brainstem injury were more strongly correlated with tracheostomy need. This suggests that in TBI patients, the need for tracheostomy due to respiratory failure is primarily tied to the severity of the brain injury itself.

Firstly, in the TBI patient cohort, significant differences were observed between individuals with and without DAI in terms of tracheostomy requirement and injury cause. DAI, a prevalent and severe TBI pathology, is characterized by extensive axonal damage due to shear forces or rotational acceleration/deceleration (42). Our study found a higher proportion of patients needing tracheostomy among those with DAI, aligning with the results of Srinivas et al. (43). This underscores the multiple complications, including tracheostomy-related issues, and generally poor prognosis faced by DAI patients (44, 45).

Further analysis reveals that, among patients with TBI who have undergone tracheotomy, there are differences between those with and without DAI in terms of the injury mechanism and history of decompressive craniectomy. This suggests that in the subgroup of severe TBI patients requiring tracheostomy, specific injury mechanisms (those more likely to cause DAI) and more severe intracranial pathology (such as severe cerebral oedema or intracranial hypertension requiring decompressive craniectomy) are associated with the presence of DAI. In TBI patients who did not undergo tracheostomy, no statistically significant differences were found between groups with and without DAI.

To deepen our comprehension of the factors associated with tracheostomy in patients with TBI, the correlation analysis in this study reveals a moderate negative correlation between the GCS score and the necessity for tracheostomy in TBI patients. Previous research has consistently shown a strong association between GCS scores below 8 and the requirement for tracheostomy (46). However, this relationship is not fixed and varies with the patient’s clinical progression (14). Therefore, it is advisable to consider the GCS score in conjunction with other factors (e.g., DAI, performance of decompressive craniectomy, pupil response) for a more precise prediction of tracheostomy necessity in TBI patients.

The history of craniectomy, the course of the disease, and age are moderately correlated with the need for tracheostomy. Craniectomy is an active surgical intervention for TBI patients, typically used for those with a low GCS score, severe basal ganglia compression, and midline shift, which require surgical support (47). These patients, due to elevated intracranial pressure and brainstem compression, require early establishment of a stable respiratory support pathway (48, 49). Furthermore, as concluded from the descriptive analysis, the impact of age on the need for tracheostomy in TBI patients cannot be ignored. Our correlation analysis further confirmed this, with a study by Chiara et al. showing that for every 5-year increase in age, the risk of tracheostomy rises by 4% (14). Some studies suggest that frailty and sarcopenia often occur simultaneously in elderly patients, with muscle dysfunction caused by sarcopenia directly affecting respiratory function and secretion clearance (50). Mubashir et al. (51) proposed that as age increases, respiratory function gradually declines in TBI patients, making airway management more complicated. However, some studies did not find an effect of age on the need for tracheostomy (52). Despite existing data showing some controversy regarding the relationship between age and the risk of tracheostomy, our clinical validation has initially confirmed the role of age in risk assessment.

Abnormal pupil responses, the Marshall CT classification score, and brainstem injury show a weak correlation with the need for tracheostomy. Pupillary response abnormalities serve as clinical warning signs of brainstem functional impairment or increased intracranial pressure (53). Changes in pupillary response following brain trauma are closely correlated with the patient’s condition severity (54). Brainstem injury is strongly associated with the need for tracheostomy. Being a vital center of the central nervous system, brainstem damage frequently leads to consciousness disorders and dysfunction of the respiratory center. Previous studies have shown that diffuse axonal injury is a common pathological feature following traumatic brain injury, particularly in the brainstem area (55–57). Dysautonomia following diffuse DAI in the brainstem can result in prolonged disturbances of consciousness (58, 59), impacting the recovery of spontaneous breathing and compromising airway protective reflexes like coughing and swallowing, thus increasing the likelihood of requiring tracheostomy (14, 60). This underscores the significance of brainstem injury in predicting the necessity for tracheostomy in TBI patients. The Marshall score assesses the extent of intracranial lesions observed on CT scans, with a focus on factors such as intracranial hemorrhage, brain edema, and midline shift (61). This score suggests that the severity of brain injury is more strongly associated with the need for tracheostomy than other clinical variables.

We believe that the necessity of tracheostomy in TBI patients is predominantly determined by the severity of the brain injury and the patient’s age, with a specific focus on the severity of the injury. Healthcare providers are advised to meticulously evaluate the extent of the brain injury, utilizing standardized scoring systems, in conjunction with considerations of patient age and imaging results, to facilitate prompt decision-making that may enhance the patient’s prognosis.

## 5 Limitations

This study is limited by a small sample size and the lack of continuous dynamic monitoring indicators. Additionally, the inclusion criterion specifying patients admitted within 30 days post-injury was not consistently met, as some patients were not assessed immediately after the injury. Consequently, data collection did not occur during the patients’ most critical phase, potentially impacting the reliability of the gathered indicators in reflecting the severity of their condition. Future prospective studies are needed to investigate the relationship between tracheotomy and brain injury as opposed to pulmonary infections, warranting further empirical validation of this hypothesis.

## 6 Conclusion

This study systematically examines the predictors of tracheostomy in patients with brain injuries, specifically TBI, utilizing bibliometric analysis, descriptive analysis, and retrospective clinical research. The findings reveal a growing body of research on tracheostomy predictors in brain injury patients, focusing on extubation failure, tracheostomy decision-making, and patient prognosis. Leading contributors in this field are the United States, China, and Italy, with prominent publishing outlets including Frontiers in Neurology and Neurocritical Care. Moreover, there are disease-specific characteristics influencing the demand for tracheostomy in brain injury patients. For patients with TBI, it is crucial to consider the etiology of the injury, the patient’s age, and assessments of brain damage severity. These results can assist healthcare providers in identifying high-risk patients early and can serve as a scientific rationale for optimizing the timing of tracheostomy, potentially enhancing clinical outcomes.

## Data Availability

The raw data supporting the conclusions of this article will be made available by the authors, without undue reservation.
