# Supplementary material for: Multistage investigation of predictive factors for tracheostomy in brain injury: a bibliometric, descriptive, and retrospective analysis
Source: Front Neurol. 2025 Sep 24;16:1648046. doi: 10.3389/fneur.2025.1648046 (PMC12504103; doi:10.3389/fneur.2025.1648046)
Supplement: Supplementary file 1 [file Table_1.DOCX]

Supplement table. 1 Details of the 5 articles included in the descriptive analysis

| **Author** | **Country and Institution** | **Years** | **Types of brain injury** | **Strong predictors** | **Age** | **Total number of patients** | **Inclusion Criteria** | **Exclusion Criteria** | **Typle of study** | **assessments** | **Interventions** | **Primary Outcome** | **Secondary Outcomes** | **Overall bias(NOS)** |
| --- | --- | --- | --- | --- | --- | --- | --- | --- | --- | --- | --- | --- | --- | --- |
| Ryne et al | USA, the R. Adams Cowley Shock Trauma Center at the University of Maryland School of Medicine | 2018 | traumatic brain injury | Number of operation room trips; Reintubation; Placement of an external ventricular drain; GCS | _ | 209 | 1.Adult traumatic brain injury (TBI) patients requiring intensive care unit (ICU) admission for ≥72 hours and mechanical ventilation for ≥24 hours. 2.Admitted to a single level 1 trauma center between January 2014 and December 2014. | Patients with life-sustaining measures withdrawn. | Single-Center Retrospective Cohort Study | Data on admission and hospitalization of TBI patients were collected, and methods such as observational study and multivariable logistic regression were used. The results showed that inpatient factors were more strongly associated with tracheostomy, and predictors such as EVD, GCS on day 5, reintubation, and the number of trips to the operating room were identified. | Patient management was in line with the guidelines of the Brain Trauma Foundation. Daily 60 - 120-minute spontaneous breathing trials (5 cm H₂O of pressure support and 5 cm H₂O of positive end-expiratory pressure) were performed for mechanically ventilated patients without medical contraindications (such as increased intracranial pressure or status epilepticus requiring sedation as the primary therapy). | The occurrence of tracheostomy. | 1.Admission factors (GCS, ISS, inpatient factors, number of operations, etc) associated with tracheostomy. 2.The association strength between different predictive models and tracheostomy. | 6 |
| Chiara et al | Europe, The CENTER - TBI study involved a large number of collaborating institutions across Europe | 2020 | traumatic brain injury | Age; GCS; Pupillary Reactivity; Thoracic Trauma; Hypoxemia | ＞ 65 | 1358 | 1.A clinical diagnosis of TBI with an indication for a brain Computed Tomography scan (CT). 2.Presentation to the hospital within 24 h (hrs) postinjury. 3.ICU admission with a length of stay (LOS) ≥72 h. | 1.Death in the first 72 h. 2.Short ICU LOS (<72 h). | Multi-Center Prospective Cohort Study | Data of TBI patients from the CENTER - TBI study were collected. Multiple regression models and methods for dealing with missing values were used. Predictors for tracheostomy such as age, GCS, and thoracic trauma were identified. Differences in tracheostomy practice among countries and centers were found. Early tracheostomy was associated with better outcomes. | 1.Tracheostomy: Performed according to clinical decisions, with some patients receiving early tracheostomy (≤7 days) and some receiving late tracheostomy (>7 days). 2.Other treatment measures included cranial surgery, extracranial surgery, placement of an intracranial pressure (ICP) monitoring device, use of antibiotics, mechanical ventilation, etc. | Functional outcome at 6 months, assessed by the Extended Glasgow Outcome Score (GOSE), with GOSE ≤4 defined as an unfavorable outcome, taking mortality into account. | 1.Patient characteristics(age, sex, race, ASAPS score, ISS, hypotension, pupillary reactivity, GCS, etc) related to tracheostomy. 2.Tracheostomy factors( timing, factors affecting the decision). 3.Differences: tracheostomy incidence and timing among countries and centers. 4.Other measures( ICU/hospital mortality, LOS, ventilation complications, and treatment details). | 8 |
| Llko et al | Germany, the University Medical Center Göttingen | 2021 | Large Vessel Occlusion Stroke | Hospital-acquired pneumonia; Sepsis; Failed extubation; Decompressive hemicraniectomy | _ | 635 | 1.Patients with large vessel occlusion stroke (LVOS) who received mechanical thrombectomy (MT) treatment in a large academic neurological intensive care unit (neuro-ICU) between 2014 and 2019. 2.Having a complete predefined dataset including pre-stroke history (comorbidities), peri-interventional data (imaging, time metrics, scores), and post-stroke clinical data (such as infectious complications). | Patients with spontaneous reperfusion or reperfusion through intravenous thrombolysis (IVT) of the occluded vessel on the first angiogram (defined as modified Thrombolysis in Cerebral Infarction (mTICI) >1). | Single-Center Prospective Cohort Study | Data of LVOS patients treated with MT in a single center were collected, using prospective (partially retrospective) and logistic regression methods. Predictors for tracheostomy such as HAP, sepsis, failed extubation, and DH were identified, and a score with good predictive value was created. | 1.All patients received mechanical thrombectomy treatment, and some patients received intravenous thrombolysis treatment according to the current German guidelines. 2.The decision of tracheostomy was made by ICU-trained senior consultant neurologists, and all tracheostomies were performed as surgical procedures by experienced Ear, Nose, and Throat (ENT) specialists. 3.The indication for decompressive hemicraniectomy (DH) was determined in consensus with stroke-experienced neurosurgeons. | Whether tracheostomy is required | 1.Patient characteristics( baseline details, clinical scores, and imaging features) related to tracheostomy. 2.Treatment characteristics( IVT, anesthesia type, and hospitalization/ventilation durations). 3.Complications( extubation failure, HAP, sepsis, ICH types, and DH.Predictive factors: identified through regression, with a created score evaluated for prediction).  4.Correlations analyzed between tracheostomy time and clinical, hospitalization, and ventilation outcomes. | 7 |
| XiaoYong et al | China, the First Affiliated Hospital of Fujian Medical University | 2021 | Aneurysmal Subarachnoid Hemorrhage | Age ≥60; High neutrophil-to-lymphocyte ratio; High World Federation of Neurological Surgeons Scale grade;High Barrow Neurological Institute grade | ≥ 60 | 488 | 1.Age＞18 years old. 2.A diagnosis of aSAH based on preoperative computed tomography angiography (CTA) or digital subtraction angiography (DSA). 3.Received surgical treatment including interventional embolization or clipping after admission. 4.Patients admitted within 48 h and did not receive intervention before admission. 5.Preoperative routine blood test was obtained in emergency room or inpatient wards within the first 2 h of admission. 6.Other medical records were complete. | 1.Patients were <18 years old. 2.Incomplete medical information. 3.Patients with previous or preoperative TT. 4.Previous use of steroids, antiplatelet or anticoagulant drugs, or immunosuppressants. 5.Combined other neurological diseases or serious diseases. | Single-Center Retrospective Cohort Study | Collected baseline, aneurysm and surgery-related information of aSAH patients, using retrospective study and multiple analysis methods. Identified age, NLR, WFNS and BNI grade as predictors, and established and validated a nomogram model with good predictive power in training and validation cohorts. | Active treatment (interventional embolization or clipping), and then the decision of postoperative tracheostomy (TT) was jointly made by neurosurgeons, intensive care unit clinicians, and otorhinolaryngologists based on neurological function, airway, age, and other factors for those patients who required artificial airway and respiratory support more than 2 weeks. | Occurrence of postoperative tracheostomy (TT) | 1.Occurrence of delayed cerebral vasospasm and delayed cerebral ischemia. 2.Evaluation metrics of the predictive ability of the model. | 4 |
| Felix et al | Germany, the University Hospital RWTH Aachen, the Cologne-Merheim Medical Centre, the University Hospital Frankfurt/Main, the Medical Centre Albert - Ludwigs - University of Freiburg, the University Hospital Schleswig - Holstein in Kiel, and the University Hospital Giessen and Marburg GmbH in Marburg. | 2023 | traumatic brain injury(Head AIS ≥3 and Thorax≥3) | Prehospital endotracheal intubation; Diagnosis of pneumonia during the ICU stay; Duration of mechanical ventilation; AISHead ≥3; Sepsis | _ | 1019 | 1.Patients aged 18 years or older. 2.Admitted to one of the six participating German academic level I trauma centres between 2010 and 2014. 3.Suffered from multiple injuries, including severe thoracic trauma (AISThorax ≥3). | _ | Multi-Center Retrospective Cohort Study | Data of adult thoracic trauma patients from six German level I trauma centers were collected. Retrospective cohort study and multivariate regression analysis methods were used. Predictors for tracheostomy were identified and the T3P -Score was developed, with high predictive validity, a specificity of 0.68 and a sensitivity of 0.96. | _ | Whether tracheostomy is required. | 1.Patient characteristics( Demographic characteristics of patients, injury-related factors, other clinical characteristics, Revised Injury Severity Score II (RISC II), mortality, use of nitric oxide, use of extracorporeal membrane oxygenation ) related to tracheostomy. 2.Determine the predictors of tracheostomy, and develop the Tracheostomy in Thoracic Trauma Prediction Score, evaluate the predictive validity of the score | 4 |

Supplement table. 2 The 10 Most Prolific Authors

| **No.** | **Author** | **Documents** |
| --- | --- | --- |
| 1 | GAMBARDELLA, IVANCARMINE | 4 |
| 2 | GIRARDI, LEONARD N. | 4 |
| 3 | LAU, CHRISTOPHER | 4 |
| 4 | CINOTTI, RAPHAEL | 3 |
| 5 | DRAGHI, FRANCESCA | 3 |
| 6 | HAKIKI, BAHIA | 3 |
| 7 | MACCHI, CLAUDIO | 3 |
| 8 | PELOSI, PAOLO | 3 |
| 9 | RABINSTEIN, ALEJANDRO A. | 3 |
| 10 | ROBBA, CHIARA | 3 |

Supplement table. 3

| **No.** | **Organization** | **Documents** | **citations** | **Average Citation/Publication** |
| --- | --- | --- | --- | --- |
| 1 | University of Toronto | 7 | 57 | 8.1 |
| 2 | Capital Medical University | 5 | 17 | 3.4 |
| 3 | University of Genoa | 5 | 108 | 21.6 |
| 4 | University Health Network | 5 | 53 | 10.6 |
| 5 | Emory University | 4 | 25 | 6.25 |
| 6 | Huazhong University of Science and Technology | 4 | 43 | 10.7 |
| 7 | Weill Cornell Medicine | 4 | 30 | 7.5 |
| 8 | Hamad Medical Corporation | 3 | 48 | 16 |
| 9 | Mahidol University | 3 | 46 | 15.3 |
| 10 | Mayo Clinic | 3 | 25 | 8.33 |
| 11 | National Taiwan University Hospital | 3 | 6 | 2 |
| 12 | University of Florence | 3 | 30 | 10 |
| 13 | University of Freiburg | 3 | 13 | 4.3 |
| 14 | University of Oxford | 3 | 46 | 15.3 |
| 15 | University of Rochester | 3 | 0 | 0 |

Supplement table. 4

| **No.** | **Journal** | **Citations** |
| --- | --- | --- |
| 1 | Stroke | 130 |
| 2 | Critical Care Medicine | 127 |
| 3 | Neurocrit Care | 97 |
| 4 | Critcal Care | 85 |
| 5 | Intensive Care Medicine | 82 |
| 6 | Journal of Thoracic and Cardiovascular Surgery | 76 |
| 7 | Journal of Trauma and Acute Care Surgeru | 69 |
| 8 | JAMA-Journal of Amercian Medical Association | 62 |
| 9 | Amercian Journal of Respiratory and Critical Care Medicine | 60 |
| 10 | Journal of Vascular Surgery | 54 |

Supplement Table. 5

| **Year** | **First author** | **Title** | **DOI** | **Citations** |
| --- | --- | --- | --- | --- |
| 2013 | Bösel, Julian | Stroke-related Early Tracheostomy versus Prolonged Orotracheal Intubation in Neurocritical Care Trial (SETPOINT): a randomized pilot trial. | 10.1161/strokeaha.112.669895 | 11 |
| 2000 | Coplin, W M | Implications of extubation delay in brain-injured patients meeting standard weaning criteria. | 10.1164/ajrccm.161.5.9905102 | 11 |
| 2011 | Pelosi, Paolo | Management and outcome of mechanically ventilated neurologic patients. | 10.1097/ccm.0b013e31821209a8 | 11 |
| 2013 | Young, Duncan | Effect of early vs late tracheostomy placement on survival in patients receiving mechanical ventilation: the TracMan randomized trial. | 10.1001/jama.2013.5154 | 10 |
| 2017 | Enrichi, Claudia | Clinical Criteria for Tracheostomy Decannulation in Subjects with Acquired Brain Injury | 10.4187/respcare.05470 | 10 |
